# A genome-wide analysis reveals that the *Drosophila *transcription factor Lola promotes axon growth in part by suppressing expression of the actin nucleation factor Spire

**DOI:** 10.1186/1749-8104-6-37

**Published:** 2011-11-30

**Authors:** Michael A Gates, Ramakrishnan Kannan, Edward Giniger

**Affiliations:** 1 Basic Neuroscience Program, National Institutes of Neurological Disorders and Stroke, National Institutes of Health, Bethesda, MD 20892, USA; 2 National Human Genome Research Institute, National Institutes of Health, Bethesda, MD, 20892 USA; 3 Fred Hutchinson Cancer Research Center, Seattle, WA, 98109 USA; 4 Graduate Program in Molecular and Cellular Biology, University of Washington and Fred Hutchinson Cancer Research Center, Seattle, WA, 98109 USA

## Abstract

**Background:**

The phylogenetically conserved transcription factor Lola is essential for many aspects of axon growth and guidance, synapse formation and neural circuit development in *Drosophila*. To date it has been difficult, however, to obtain an overall view of Lola functions and mechanisms.

**Results:**

We use expression microarrays to identify the *lola*-dependent transcriptome in the *Drosophila *embryo. We find that *lola *regulates the expression of a large selection of genes that are known to affect each of several *lola*-dependent developmental processes. Among other loci, we find *lola *to be a negative regulator of *spire*, an actin nucleation factor that has been studied for its essential role in oogenesis. We show that *spire *is expressed in the nervous system and is required for a known *lola*-dependent axon guidance decision, growth of ISNb motor axons. We further show that reducing *spire *gene dosage suppresses this aspect of the *lola *phenotype, verifying that derepression of *spire *is an important contributor to the axon stalling phenotype of embryonic motor axons in *lola *mutants.

**Conclusions:**

These data shed new light on the molecular mechanisms of many *lola*-dependent processes, and also identify several developmental processes not previously linked to *lola *that are apt to be regulated by this transcription factor. These data further demonstrate that excessive expression of the actin nucleation factor Spire is as deleterious for axon growth *in vivo *as is the loss of Spire, thus highlighting the need for a balance in the elementary steps of actin dynamics to achieve effective neuronal morphogenesis.

## Background

Transcriptional regulation is central to the development of neural connectivity [1-4]. First, by controlling the identities and differentiation of individual neurons, and of the non-neuronal cells that often provide them with growth and guidance cues, it specifies patterns of axon growth and dendritic ramification. For example, classic homeotic selector genes produce segment-specific patterns of axon projection by controlling transcription of key cell fate and guidance molecules [5,6]. At a higher level of specificity, the axonal projections of many individual neurons and classes of neurons are determined by the expression of particular combinations of transcription factors. Perhaps the best-characterized example of this is the 'code' of LIM domain transcription factors that controls the dorsal versus ventral projection of motoneurons from the central nervous system (CNS) in both vertebrates and invertebrates [7-9]. Thus, in *Drosophila*, for instance, the Lim/Islet/Eve-dependent pattern of motor axon projection arises in part from regulated transcription of a critical axon guidance receptor, the Netrin receptor Unc5 [10]. In addition to specifying axon projection by defining cell identity *per se*, transcriptional regulation is also key to determining more local properties of axon patterning. For example, recent experiments have shown that the attractive midline receptor DCC in addition to controlling cytoplasmic signal transduction and growth cone motility directly, further contributes to midline axon guidance by inducing expression of *commissureless *mRNA [11].

Beyond simply expressing the right proteins, however, a neuron must express these proteins at the proper relative levels such that it responds appropriately to precise ratios of specific external and internal cues [12-16]. Again, tight regulation of gene expression is essential to make this happen. The *Drosophila *transcription factor gene *lola *is an exemplar of this latter role of transcriptional regulation. Thus, for example, *lola *orchestrates midline crossing by CNS axons by coordinately regulating the expression of both the midline repellant Slit and also its axonal receptor, Robo [17]. The effects of *lola *on *slit *and *robo *expression individually are modest, but in combination, the cumulative effect is profound, and further enhanced by *lola*-dependent regulation of other components of the pathway (*robo2 *and *robo3*; HJ Song, BJ Taylor and EG, unpublished observations). Similarly, detailed analysis of mutant phenotypes suggested that *lola *is likely to be a 'master regulator' of the patterning of the ISNb motonerve of *Drosophila*, coordinately regulating expression of the suite of guidance proteins responsible for ISNb motoneuron/muscle interaction [18].

In addition to its well-characterized functions during early embryonic axonogenesis, *lola *is also involved in construction or function of neural circuits throughout the lifespan of the fly. This includes dendritic morphogenesis in embryonic sensory neurons [19] and both dendritic and axonal development of adult olfactory projection neurons [20]. Moreover, *lola *has been identified in a number of genetic and genomic screens for genes involved in complex behaviors, including locomotion, alcohol sensitivity, mating and aggression [21-24]. While the role of *lola *in these processes is less well understood than is its well-studied function in axon patterning, the varied effects of *lola *underscore its importance for establishing patterns of gene regulation that produce a properly functioning nervous system. Beyond its neuronal functions, *lola *also plays key roles in several non-neuronal tissues. For example, *lola *is essential for oogenesis, in part due to its regulation of programmed cell death at different stages of oogenesis [25].

The *lola *locus encodes a family of more than 20 transcription factor isoforms by alternative splicing [26-28]. All share a BTB dimerization domain at the amino terminus, allowing co-expressed Lola proteins in a cell to form a variety of heterodimeric species *in vivo*. At least 17 *lola *isoforms encode Zn fingers in their variant 3' exons. While some isoforms have a single Zn finger, in most isoforms, the Zn fingers are present as a pair, with an amino-terminal CCHC Zn finger that is apt to be a protein interaction domain, followed by a C2H2 Zn finger that is likely to bind nucleic acid [26]. Biochemical experiments reveal that isoform K binds directly to the transcriptional enhancer of the *copia *retrotransposon, activating its expression in transient transfection assays [29]. A different isoform, isoform T, has a nearly identical sequence as isoform K in its Zn fingers and binds the same DNA sequence, but it suppresses *copia *expression, competing with isoform K in co-transfection experiments. Consistent with these evidently antagonistic effects of *lola *isoforms, expression of the endogenous *copia *is upregulated in the CNS of *lola *mutants, but downregulated in the gonads of the same individuals.

To better understand the regulation of axon growth and guidance by *lola*, we performed a microarray study to identify genes whose transcript level was altered in a *lola *mutant. In addition to the sole known direct target of Lola, the retrotransposon *copia*, we found changes in expression level of a substantial number of genes that have been implicated previously in well characterized *lola*-dependent developmental events, including axon patterning, eye development, *Notch *signaling and programmed cell death. Among the affected genes was an unexpected downstream target, the gene encoding the actin nucleation factor Spire, which was upregulated in the *lola *mutant. *spire *is required for both anteroposterior and dorsoventral patterning of the developing oocyte [30], but its zygotic functions have never been characterized. We found that mutation of *spire *by itself caused defects in a *lola*-dependent axon patterning event, extension of the ISNb motonerve, and further that reduction of *spire *expression, by introduction of a heterozygous *spire *mutation, suppressed the axonal defects of *lola *in extension of the ISNb motonerve. These data suggest that overexpression of *spire *makes a significant contribution to the ISNb axonal phenotype of *lola*.

## Results

### Design of the microarray experiment

We set out to use cDNA microarray analysis to profile the changes in gene expression in *lola *mutant embryos relative to wild type. For detailed protocol see Materials and methods and [31], but key points are as follows. Since our primary interest was in axon patterning, we collected embryos at 10 to 12 hours after egg laying, a time when many axons are extending along *lola*-dependent nerve pathways in the embryo. We employed a molecular-null *lola *allele, *lola^ORE76^*, which introduces a stop codon early in the common region of *lola *and does not accumulate detectable levels of any Lola protein fragment *in vivo*. Seven pairs of mutant and control wild-type RNA samples were collected independently, with each sample derived from approximately 300 embryos, and each pair collected in parallel on the same day. Homozygous mutant embryos were positively selected in the fluorescent microscope using *GAL4*-driven expression of a green fluorescent protein (*GFP*) marker in the mutants, and controls were selected in a parallel cross employing the same *GAL4 *and *GFP *markers. Labeled first-strand cDNA programmed from experimental and control samples were co-hybridized to spotted cDNA arrays bearing the *Drosophila *Gene Collection (release 1 and release 2), prepared for the Northwest *Drosophila *Microarray Consortium (Gene Expression Omnibus Platform GPL 1908) [32,33]. For four pairs of samples, the experimental probe was labeled with Cy-3 and control with Cy-5, while dye assignments were reversed for the remaining three sample pairs.

**Figure 1:**
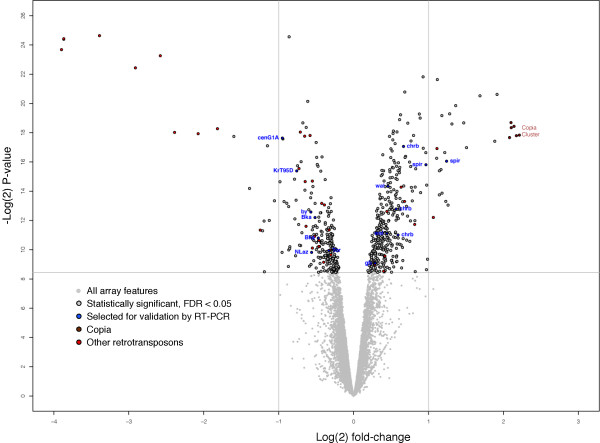
**Distribution of effects on transcript accumulation in *lola *microarrays**. Graphical representation of data from *lola *microarray experiment. Each gray dot represents the mean ratio of normalized feature hybridization for *lola *mutant/wild type from seven experiments, presented as a scatter plot of log2(adjusted *P*-value) versus log2(fold change) for each cDNA. Points above the horizontal line (circled in black) are statistically significant with the false discovery rate controlled to 0.05 (which, for this analysis, roughly corresponded to adjusted *P *< 0.0028). The six cDNAs corresponding to the *copia *retrotransposon are highlighted in brown; other retrotransposon transcripts are in red. Features corresponding to loci also quantified by quantitative RT-PCR are in blue. Note that different cDNAs corresponding to a single locus are plotted separately; thus, for example, there is a cluster of six, independent brown spots for *copia*.

*Drosophila *arrays had 12, 144 features (that is, cDNA spots), of which 10, 376 were analyzed statistically for differential expression. Data processing and statistical analysis were performed using the *limma *statistical package [34,35]. Standard errors were modified using an empirical Bayes method as implemented in *limma*. Adjustment for multiple testing was performed by controlling the false discovery rate to 5% [36]. Selection of features as showing differential expression was based solely on statistical significance: no *a priori *assumptions were made as to minimum expression level or minimum fold-change in expression. This analysis identified 597 genes that showed differential expression between mutant and control (displayed graphically in Figure 1; complete list in Table S1 in Additional file 1), with significant fold changes as low as 1.14-fold.

### Expression of *copia *and other retrotransposons is altered in *lola *mutants

Inspection of the features that passed our analysis revealed that six among those with the largest fold change (4.2 to 4.6) corresponded to six independent cDNAs of the retrotransposon *copia*. *copia *is the only verified direct target of Lola, and bears Lola binding sites in its enhancer. *lola *isoform K activates *copia *expression in cultured cells, and *in situ *hybridization reveals that *copia *RNA levels are altered in *lola *mutant embryos [29]. Quantitatively, all six features corresponding to *copia *showed a similar factor of increase in expression level in the mutants, and these six cDNAs were the only features on the array that corresponded to *copia*. In addition to *copia*, we also detected significant expression changes in features corresponding to several other retrotransposons, including *stalker*, *stalker2*, *297 *and *roo*. Whether these are also direct Lola targets or indirectly dependent on *lola *remains to be examined.

### Validation of positives from the array analysis

Two lines of evidence support the validity of the positives from the array analysis. First, we performed quantitative RT-PCR (qRT-PCR) of ten genes with statistically significant changes in expression level between mutant and control. To give the best assessment of the overall reliability of the dataset, we selected genes that showed a range of fold change (1.2 to 2.4), and we excluded retrotransposons, which showed the largest fold changes in the dataset (Figure 1). Of the ten genes selected, five were validated in the qRT-PCR as changed in level in RNA samples from the *lola *null mutant (*P *< 0.05; Table 1). We suggest that this provides a minimum estimate of the reliability of the microarray dataset for two reasons. First, the cDNA representing a particular gene on the array and the associated qRT-PCR target in the validation experiment may not query the same splice variant or set of splice variants, and thus may be regulated differently. Second, the RNA for the array analysis was derived, in aggregate, from 2, 100 embryos per genotype while the qRT-PCR samples were derived from 150 to 250 embryos. The substantially larger size of the sample contributing to the array analysis, therefore, may have materially reduced its variance compared with the qRT-PCR.

**Table 1:** Quantitative RT-PCR validation of array results

Gene	Clone ID	Array fold-change (log2)	qRT-PCR fold-change (log2)	qRT-PCR *P*-value
*spire*	GH13327	1.24	1.54	2.5 E-5
	SD10157	0.97		
*neural lazarillo*	GH12581	-0.56	-2.40	9.3 E-5
*genderblind/CG6070*	GH08870	0.29	0.27	0.016
*charybde*	LD32080	0.57	0.33	0.036
	GH09771	0.59		
	LD22381	0.67		
*walrus*	GH09945	0.46	0.27	0.039
	LD07532	0.44		
*centaurin gamma 1a*	GM01069	-0.95	-0.13	0.25
*blistery*	SD01953	-0.57	0.22	0.34
*bekka*	RE14959	-0.47	-0.46	0.61
	RE14259	-0.52		
*Kr target @ 95D*	SD03976	-0.76	0.03	0.79
*Target of rapamycin*	SD02269	-0.33	0.33	0.95

Total RNA was collected from 10 to 12 hour wild type or *lola *mutant embryos, just as for microarray analysis, and analyzed by Taqman RT-PCR to quantify expression of ten genes that showed altered transcript level in expression arrays. A positive fold change in the table corresponds to increased accumulation of that RNA in the mutant.

**Figure 2:**
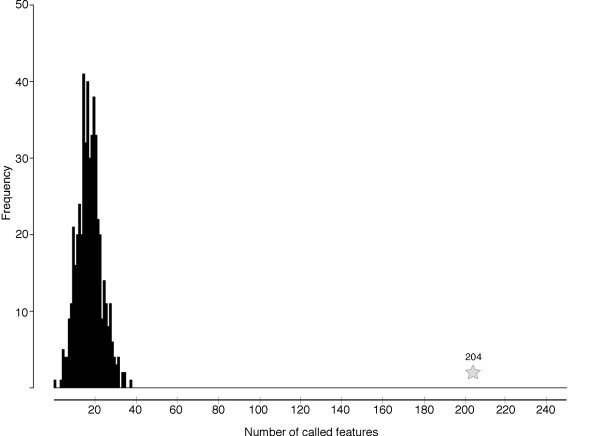
**Validation of *lola *null data with an independent microarray experiment**. Graphical representation of an experiment assessing the validity of the *lola *null mutant results by comparison to a dataset derived from an independent microarray experiment. Expression microarrays were performed comparing RNA from embryos bearing the *lola *single-isoform mutation *lola^ORC4 ^*to wild-type controls [31]. The 597 features that were altered in accumulation in *lola *null mutant embryos were assessed for altered expression in *lola^ORC4^*: 204 of the 597 were also found to be altered in the single-isoform mutant (false discovery rate = 0.01; grey star). In contrast, in 500 trials of a simulation in which 597 features were selected at random from the *lola^ORC4 ^*data set (10, 376 features total) and analyzed for change in mutant versus wild type, the median number of features with altered expression in the single-isoform mutant was 18/597 (maximum 38; presented as a histogram of number of loci with altered expression versus number of occurrences of that result).

As a second validation, the results of the array analysis were also supported by an independent microarray experiment. Expression profiling was performed for a different kind of *lola *mutation, the allele *lola^ORC4 ^*that inactivates only a single *lola *isoform, *lola K *[26,31]. When we examined the expression profile of *lola^ORC4 ^*mutant embryos versus their matched control samples, and limited our statistical analysis to the set of 597 features with significantly altered expression in the *lola *null mutant, we found that 204 of these features also showed differential expression in the *lola^ORC4 ^*dataset (false discovery rate controlled at 0.01; Figure 2). In contrast, in 500 simulations in which 597 features were selected at random from the *lola^ORC4 ^*dataset (out of the 10, 376 features queried in the arrays), the median number that showed an expression change in *lola^ORC4 ^*was 18 (maximum 38). Thus, the set of features identified as *lola*-dependent in the null mutant sample provided a substantial enrichment of *lola*-sensitive features compared to the complete gene set, as assayed in an independent microarray experiment. This strongly supports the validity of the positives called in the original microarray analysis of the null.

Therefore, the combination of qRT-PCR of selected hits and a global validation by an independent array experiment provides strong evidence for the reliability of the identification of *lola*-sensitive transcripts by the microarray analysis.

### Gene Ontology analysis of transcripts altered in a *lola *null mutant

The complete list of expression effects of *lola *revealed 597 features with altered labeling out of the 10, 376 features that were assayed in the experiment. Of these, 352 were increased in level in the *lola *mutant, implying direct or indirect negative regulation, while 245 were decreased. Consistent with the broad expression of *lola *itself, these features represent genes expressed in a wide variety of tissues and at all stages of the lifecycle. Gene Ontology analysis [37], however, identifies a large number of previously characterized genes that are associated with known *lola*-dependent processes, including cell and axon migration and orientation, eye development, oogenesis and programmed cell death (Table 2). This analysis also reveals potential effects of *lola *on a variety of developmental and physiological processes for which such a role had not previously been suspected, including spermatogenesis, epithelial polarity, hormonal regulation of development, oxidative stress and aging/lifespan (Table 3).

**Table 2:** Selected set of *lola*-dependent genes from microarray analysis

**Axon patterning**
*frazzled*
*DSCAM*
*neural lazarillo*
*Fasciclin III*
*midline fasciclin*
*prospero*
*capricious*
*PVF-1*
*CBP (sarcoplasmic Ca binding prot)*
*sugarless*
*dally-like*
**Cell and tissue polarity**
*par-1*
*par-6*
*scribble*
*wnt-4*
**Signaling proteins**
*Cask (CaM kinase)*
*moesin*
*rhoGAP 5A*
*Target of Rapamycin*
*rac2*
**Microtubules and motors**
*katanin80*
*stathmin*
*KLP-59C*
*NudC*
**Eye development**
*sickle*
*charlatan*
*asense^a^*
*scabrous^a^*
*rap1*
*roughex*
*Dap160^a^*
*Lobe*
*twin of eyeless*
*fat facets*
*numb-associated kinase^a^*
*O-Fut-1^a^*
**Cell death**
*grim*
*scylla*
*charybde*
*bunched^a^*
*Nedd2-like caspase*

Analysis of the list of loci with altered expression in a *lola *mutant reveals a substantial number of characterized genes with known or plausible function in established *lola*-dependent processes. A selection of such loci is presented here. *^a^*Genes known to be associated with *Notch *signaling.

**Table 3:** Enriched Gene Ontology categories from functional annotation clustering

Functional annotation	Enrichment factor
PDZ domain-containing proteins	1.7
Aging/lifespan	1.65
Cell migration/motility	1.65
Nucleoside/nucleotide metabolism	1.55
Reactive oxygen response; oxidative stress	1.27
BESS/MADF chromatin modifying factors	1.24
Programmed cell death	1.24
Amino acid metabolism	1.22
LRR-containing proteins	1.2
GST activity	1.18
Cell-cell junctions/apicobasal polarity	1.13
Malate metabolism	1.01
**Other notable annotation clusters**	
Embryonic morphogenesis	0.82
Chemosensory behavior/olfactory learning	0.8
Hemopoiesis/hemocyte migration	0.78
Synaptogenesis/synapse organization	0.77
Post-embryonic development/imaginal disc	0.7
Gametogenesis	0.54
Spermatogenesis	0.3
Oogenesis	0.26
Ig-like domain-containing	0.4
Axonogenesis/neuron morphogenesis	0.28
RNPs	0.27

Gene Ontology analysis with DAVID [37] was used to identify processes and properties likely to be associated with *lola *function, based on enrichment in the collection of *lola*-sensitive genes. Putatively enriched annotation terms and their enrichment factors are shown, derived by functional annotation clustering performed at high stringency setting. Clusters with enrichment factors ≥1.3 are supported at *P *< 0.05; lower enrichment scores have proportionately lower statistical support but nonetheless are enriched in the dataset relative to background. The background set for calculating enrichment factors consisted of that subset of features on the microarray that passed the pre-processing steps and entered our statistical analysis (10, 376 features), and that could be assigned unambiguously to a single Flybase gene ID (9772 Flybase-defined genes). Retrotransposons were excluded from this analysis.

### Overexpression of the actin nucleation factor Spire is an important contributor to the *lola *ISNb axon stalling phenotype

Aside from retrotransposon transcripts, one of the most robust positives in the array analysis was *spire*. *spire *encodes a protein that nucleates assembly of actin filaments [38], and extensive studies of its role in oogenesis have shown that it plays a central role in controlling cytoskeletal organization in the developing oocyte [39,40]. It is clear that *spire *also has essential roles during development since most homozygous *spire *mutants do not survive to adulthood, but these developmental functions of *spire *have never been reported. Moreover a mouse ortholog of *spire *is expressed in the developing CNS [41]. Given the action of *spire *in a different *lola*-regulated process and our interest in the regulation of neuronal actin organization by *lola*, we elected to test further the relationship of *lola *to *spire*.

**Figure 3:**
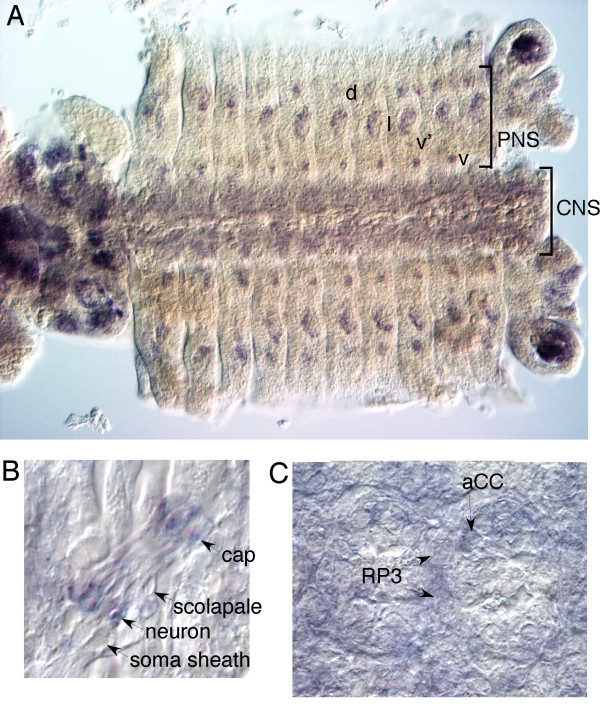
***spire *expression in the central nervous system and peripheral nervous system of *Drosophila *embryos**. Wild-type embryos were collected, fixed, and analyzed by *in situ *hybridization with a probe that recognizes all *spire *isoforms, using alkaline phosphatase detection. Widespread expression of *spire *was observed in both the peripheral nervous system (PNS) and CNS of wild-type embryos. We did not discern any difference in the pattern of hybridization in *lola *mutant embryos. We also did not detect any difference in the strength of the *spire *signal, though this is not surprising given the small magnitude of the effect. **(A) **Filet preparation of entire embryo (stage 14). PNS and CNS are indicated with brackets. Dorsal, lateral, ventral' and ventral clusters of peripheral sensory organs are indicated as d, l, v' and v, respectively. Anterior is to the left. **(B) **High magnification view of a lateral sense organ cluster (stage 16) showing preferential expression in the neurons and cap cells of the chordotonal organs. Soma sheath and scolopale cells did not label detectably. **(C) ***spire *RNA was detected in many cells of the CNS. The motoneurons RP3 and aCC are indicated (arrows). Two segments are shown.

We first used *in situ *hybridization and found that *spire *is expressed broadly in the embryonic central and peripheral nervous system of the fly (Figure 3). In the CNS, expression included the RP, aCC and VUM motoneurons, which are easy to identify individually based on their position and morphology, as well as many other cells. In the peripheral nervous system (PNS), expression was detected in all four clusters of abdominal sense organs, with highest expression in the neurons and cap cells of the chordotonal organs, and lowest expression in the v' cluster.

**Figure 4:**
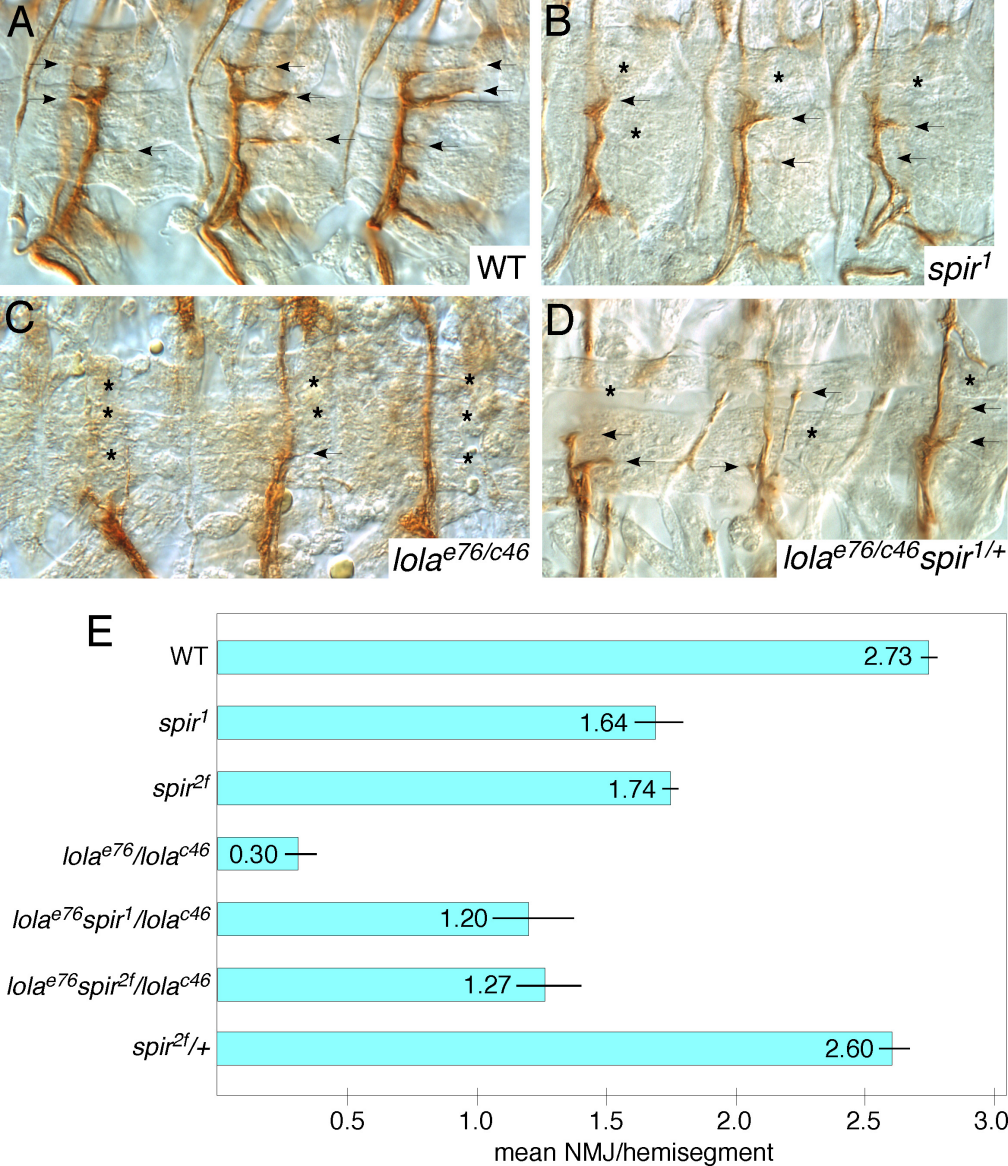
**Genetic interaction of *lola *and *spire *mutations**. Stage 17 embryos of the indicated genotypes were fixed and stained with anti-Fasciclin 2 to label ISNb motor axons, and visualized by peroxidase histochemistry. Three representative hemisegments are shown in each panel. **(A) **Wild type (WT). ISNb forms three neuromuscular junctions (NMJs) to ventral longitudinal muscles per hemisegment (arrows). **(B) ***spir^1 ^*homozygous mutant. NMJs are indicated with arrows, positions where NMJs are missing are highlighted with asterisks. **(C) ***lola^e76^/lola^c46 ^*null mutant. **(D) ***lola^e76^spir^1^/lola^c46 ^*embryo. Note partial restoration of NMJs. **(E) **Quantification of NMJ number in embryos of the indicated genotypes. Average number of NMJs per hemisegment is plotted as blue bars. Thin lines indicate standard error of the mean of three experiments (N = approximately 200 hemisegments per dataset).

We next examined neural development in *spire *zygotic mutants and found that while the CNS axon scaffold appeared grossly normal, axons of the ISNb motonerve often failed to fully innervate the ventrolateral musculature (Figure 4). Thus, in stage 17 *spir^1 ^*mutant embryos we detected 1.64 ± 0.09 (mean ± standard error of the mean) neuromuscular synapses to ventral longitudinal muscles per hemisegment versus 2.73 ± 0.03 in wild-type (*P *= 3E-4), while in *spir^2F ^*we detected 1.74 ± 0.03 synapses (*P *= 2.9E-5).

Since both *spire *and *lola *mutants displayed defects in ISNb, we used this phenotype to test for genetic interaction between these loci. In particular, since the array and qRT-PCR analyses showed *lola *to be a negative regulator of *spire*, we wondered whether reduction of *spire *gene dosage would suppress the *lola *mutant phenotype. We again quantified the stalling of ISNb by counting the number of neuromuscular synapses formed on ventral longitudinal muscles in early stage 17 embryos.

Heterozygosity for either of two independent *spire *alleles significantly suppressed the *lola *ISNb phenotype (Figure 4E). In a heteroallelic combination of two strong *lola *mutations (*lola^ORC46/ORE76^*) we detected an average of 0.30 ± 0.06 neuromuscular synapses per hemisegment, compared to 2.73 in wild-type embryos of this stage. Introduction of *spir^1^/^+ ^*increased that to 1.20 ± 0.15 synapses (37% suppression of the phenotype; *P *= 0.005), while *spir^2F^/^+ ^*increased it to 1.27 ± 0.12 synapses (40% suppression; *P *= 0.002). This supports the hypothesis that the approximately two-fold overexpression of *spire *we observe in *lola *mutants contributes significantly to its motoneuron stalling phenotype. The *lola^- ^spir^-/+ ^*embryos sometimes looked a bit disorganized compared to other genotypes, but the significance of this is unclear.

## Discussion

The transcription factor Lola is required for a variety of axon growth and guidance events in the developing fly embryo [42,43]. Expression microarray analysis of *lola *mutant embryos now reveals that, rather than producing large changes in the levels of a restricted number of major-effect downstream targets, Lola appears to exert its influence via the cumulative effects of small, quantitative changes in a broad spectrum of downstream loci. One key Lola target is *spire*, which encodes an actin nucleation factor [38] that has been studied intensively for its role in regulating cytoskeletal structure in the developing fly oocyte [39,40]. *spire*, like *lola*, is required for development of ISNb motor axons, its level goes up in *lola *mutants, and reduction of *spire *dosage suppresses, but does not eliminate, the ISNb mutant phenotype of *lola*.

Previous analysis of candidate genes implicated in various *lola*-dependent axon guidance processes identified several whose expression was subtly affected by *lola*, but none that were dramatically altered. This and other observations led to the proposition that Lola might execute its effects by fine-tuning the expression levels of genes that contribute quantitatively to various guidance decisions, and not simply by turning these genes ON versus OFF [17]. As an unbiased test of this hypothesis, we used expression microarrays to perform a genome-wide comparison of the embryonic transcriptome of wild-type and *lola *zygotic mutant embryos. We analyzed RNA isolated from animals 10 to 12 hours after egg laying, at a time when a large number of *lola*-dependent axons are extending. By this analysis, the expression of no single-copy *Drosophila *gene was altered more than four-fold by *lola*, and few were altered more than 2.5-fold. It is possible that this is an underestimate due to the compression of expression ratios in microarray experiments, but qRT-PCR results were largely consistent with the array data. It is also possible that expression of some genes may have been altered by a greater factor in just a small subset of expressing cells, but we note that most *lola *isoforms are themselves expressed very broadly, making this possibility less likely [26]. Finally, we know that some genes can be affected oppositely by different *lola *isoforms, or in different tissues [29], so it may be that a small net change in expression of a *lola *target gene hides larger but counteracting changes in different cells. Nonetheless, it remains that a genome-wide analysis failed to identify any single major-effect *lola *target that would account for the *lola *axonal phenotypes. It is also true that there is a substantial maternal contribution of Lola to the embryo [42], and this may limit the measured effect of the mutation on downstream targets. We note, however, that it is the zygotic mutant phenotype of *lola *that we are seeking to explain, and it is therefore the quantitative effect of that zygotic mutant that is the relevant measurement for investigating the phenotype.

Microarray analysis has been widely used to identify genes associated with, or responsible for, many developmental and physiological processes. Typical analyses of expression microarray data emphasize genes whose level is strongly altered by the biological manipulation, often setting numerical cutoffs for change in expression level, together with statistical criteria, to identify true positives. In our experiments, we were compelled to eschew the use of a quantitative cutoff in fold change; for example, a commonly used criterion of a two-fold minimum change would have excluded from analysis all but 26 single-copy genes in the genome. Rather, the nature of the biological process we studied, and the nature of *lola*, required that we minimize the biological and technical variance to achieve exceptionally tight statistics. In the end, qRT-PCR validation of expression changes from 1.2-fold (*genderblind*) to 2.5-fold (*spire*) provided support for 50% of the putative downstream effects of *lola*. We note that this is likely to be an underestimate of the reliability of the array results since at these small fold-differences we were at or beyond the usual sensitivity of RT-PCR itself, and it is as likely that RT-PCR was reporting false negatives as the microarrays were reporting false positives. Validity of the results was also supported more globally by independent expression profiling of another *lola *allele. Thus, these data underscore the efficacy of microarray analysis for detecting reliably even quite small changes in expression level.

Known genes whose expression was altered in the *lola *mutant shed light on many *lola*-dependent processes. Previous experiments had led to the notion that *lola *likely co-regulates a suite of interacting genes that are important for particular axon guidance decisions, and indeed, we find expression of a number of well-characterized guidance receptors to be altered in *lola*. *frazzled*, which was identified as a downstream target of *lola*, is on its own known to be required for three *lola*-dependent axonal processes: ISNb development in the periphery, and both longitudinal and commissural axon extension in the CNS [44]. Among other factors downstream of *lola *are *midline fasciclin *(longitudinal and commissural axons) [45], *fasciclin 3 *and *capricious *(ISNb) [46,47] and *neural lazarillo *(thought to be involved in both longitudinal and commissural axon guidance) [48]. Also identified were genes for a number of ligands, receptors and receptor-modifying proteins not previously associated with *lola*-dependent processes, such as *sugarless*, *dallylike*, *wnt4a *and *PVF-1*. It now becomes interesting to investigate the potential role of these genes in axon patterning, and in migration and orientation of sensory neurons. Aside from cell surface and extracellular proteins, expression of genes encoding a number of intracellular signaling proteins was found to be altered, including *prospero *(which in hypomorphic alleles produces a phenotype very similar to that of *lola *(EG, unpublished observations)), as well as *moesin*, *Rac2*, and a calmodulin-dependent protein kinase (*CAKI*). An unexpected cluster of downstream effects comprised genes for proteins modulating microtubule structure and function, including *katanin*, *stathmin*, *NudC *and *KLP-59C*. *lola *also interacts genetically with the axon patterning function and other activities of the receptor *Notch *[49,50] (EG, unpublished observations), and we find a cluster of affected genes that modulate Notch action, including *sca*, *Nak*, *Dap-160 *and *O-fut1*. In addition to these known genes, Gene Ontology analysis [37] identifies a large number of *lola*-dependent loci that have not yet been characterized in the fly, but whose annotations cluster with *lola*-dependent genes of known function. This provides a substantial list of excellent candidates for additional contributors to *lola*-dependent processes. Unfortunately, the large number of Lola isoforms, and their heteromeric combinations, makes it impossible for us to extract Lola binding site consensus sequences from these candidates using standard computational approaches. Extensive molecular experiments will be necessary in the future to identify response elements for individual heteromeric forms of Lola.

*lola *has several characterized functions outside of axon patterning. For example, it affects cell fates in the eye [50], and indeed, there is a substantial group of eye patterning genes included in the list of *lola*-affected loci (*sickle*, *charlatan*, *asense*, *rap*, *roughex*, *Lobe*, *target of eyeless *and *fat facets*). Additionally, consistent with the role of *lola *in controlling programmed cell death during oogenesis [25], we find *grim*, *scylla*, *charybde*, *bunched *and *Nedd2-like caspase *among the downstream effects. It should be noted that since our microarray analysis was performed only with mid-stage embryos, we cannot distinguish whether the effects of *lola *on these postembryonic processes are mediated by the same downstream targets that we see affected during embryogenesis. Seeing that these genes can be modulated by *lola *at one stage of the lifecycle, however, makes them more attractive candidates for analysis at other stages. Finally, in addition to genes affecting known *lola*-dependent processes, the set of genes altered in *lola *mutants identifies clusters associated with new processes that would be worth investigating for a role of *lola*. These include aging, oxidative stress, hormonal regulation of development, tracheal development and maintenance, cell polarity and olfactory learning, among others.

One of the most robust putative downstream effects we identified for *lola *was downregulation of the actin nucleation factor Spire. This was immediately striking since *spire *is known to be a critical regulator of the oocyte cytoskeleton during *Drosophila *oogenesis. *spire *is required for both anteroposterior and dorsoventral patterning of the developing oocyte [30]. By modulating actin structure, Spire restrains bundling of oocyte microtubules, thereby blocking cytoplasmic streaming in the oocyte until critical antero-posterior and dorso-ventral polarity cues become stably bound to cortical anchoring sites or initiate irreversible signaling cascades [39,40]. At the biochemical level, Spire nucleates actin filaments by bringing together actin monomers to assemble a filament nucleus [38], and it may then transfer this nucleus to the associated formin, Cappuccino, which stimulates filament growth [51]. While the developmental function of *spire *has been studied most thoroughly in the oocyte, strong mutations in this gene are largely lethal, with only small numbers of escapers surviving to adulthood, and this suggested the existence of as yet uncharacterized zygotic functions of *spire*. Moreover, a mouse ortholog of *spire *is expressed in the developing and adult brain [41]. Here we found that *spire *is required for a well-characterized *lola*-dependent neuronal process, extension of the ISNb motonerve. ISNb was an ideal candidate for the sort of function we had previously hypothesized for *lola*, since it is known to depend on the summed, quantitative effects of a large collection of regulators. We therefore exploited ISNb to examine more carefully the potential interaction of *lola *and *spire*, and found that genetic reduction of *spire *suppressed the ISNb mutant phenotype of *lola*, consistent with the upregulation of *spire *in a *lola *mutant making a significant contribution to ISNb axon stalling in *lola*. By itself, expression analysis cannot distinguish whether *spire *is a direct target of Lola or whether the upregulation of *spire *message is a downstream consequence of other changes set in motion by *lola*. Further biochemical studies of the DNA binding properties of Lola isoforms will be necessary to assess this. Finally, *lola *has other phenotypes that are not suppressed by reduction of *spire*. These may reflect, for example, roles of *lola*-dependent guidance molecules that are themselves *spire *-independent, or the action of Spire-independent aspects of growth cone signaling.

Efforts to mimic the *lola *ISNb phenotype by overexpression of *spire *were not successful. There are several possible reasons for this. First, there are thought to be at least eight Spire protein isoforms, based on cDNA and expressed sequence tag data (Flybase), and it may be that a particular combination of isoforms, or a specific ratio of expression levels of different isoforms, is necessary to give the ISNb stalling phenotype. Alternatively, it may be that this phenotype is produced only when *spire *upregulation occurs in the context of some other downstream effect(s) of *lola*. Additional experiments will be necessary to discriminate among these models.

Superficially, it seems remarkable that complete loss of *spire *causes stalling of ISNb axons, yet the upregulation of *spire *that occurs in a *lola *mutant also contributes to ISNb stalling. Evidently, excessive nucleation of actin filaments from *spire *overexpression is as detrimental to growth cone motility as is the failure of actin nucleation from absence of the protein. We and others have observed similar non-linearity in the effects of a number of signaling and cytoskeletal regulatory proteins in other axon guidance paradigms, and it appears to be a common feature of the relationship of signaling to morphogenesis. Thus, for example, even though Abl tyrosine kinase pathway signaling appears to be essential for most axon growth [52], extension of longitudinal pioneer axons of the fly CNS requires suppression of Abl signaling to achieve the proper balance in the steps of actin dynamics [53]. Similarly, for the Rac GTPases, expression of dominant negative and dominant constitutive forms of the protein are equally effective for inhibiting axon motility, but in one case from excessive stabilization of actin filaments and in the other from insufficient stabilization [54]. Spire now provides another example of this generalization, and underscores the need for signaling networks to evoke a balance in the steps in actin dynamics, thus optimizing throughput through the mechanical cycle of growth cone motility [55].

*lola *mutants have profound effects on axon patterning, even though systematic molecular analysis reveals only subtle modulation of downstream target gene expression. This observation highlights the exquisite sensitivity of motility and guidance to the balance among cell signaling networks, and thus also to the gene expression mechanisms that set the boundaries of that balance.

## Conclusions

We have used a genome-wide analysis to identify the suite of genes whose expression is altered in embryos lacking the *Drosophila *transcription factor Lola. Gene Ontology analysis sheds light on the regulation of several characterized *lola *functions, including axon guidance, synapse formation, eye development and oogenesis, by revealing the *lola*-dependence of genes known to be involved in those processes, and also by identifying a large number of previously uncharacterized *lola*-dependent genes that are likely to contribute to these processes. Additionally, these results identify novel processes that are likely to be regulated by *lola*. Regarding axon patterning, our analysis reveals that Lola suppresses expression of the actin nucleation factor Spire, and this is crucial for its ability to promote growth of motor axons *in vivo*. These data underscore the critical importance of ensuring the correct levels of actin regulatory proteins in a cell to promote motility effectively.

## Materials and methods

### Genetics

Fly genetics and husbandry followed standard methods. *spire *mutant alleles were obtained from the Bloomington *Drosophila *Stock Center. For RNA extraction, mutant embryos were *w^1118^; lola^ORE76 ^sca-GAL4/lola^ORE76 ^UAS-τ-eGFP*; controls were *w^1118^; sca-GAL4/UAS-τ-eGFP*. Numbering of *lola *isoforms follows established nomenclature [26,27]. We note that since the same *lola *allele was used to generate both the *sca-GAL4 *and *UAS-τ-GFP *recombinant chromosomes, we cannot exclude the possibility that some of the gene expression effects we observed are due to modifier loci on the *lola *chromosome.

### Embryo collection and RNA preparation

Embryos were collected for 2 hours at 25°C on grape juice agar plates and incubated an additional 6 hours at 25°C in a moist chamber. Embryos of the desired genotype were then hand-sorted with a fluorescent dissecting microscope based on positive GFP expression in the CNS. Sorted embryos were returned to 25°C and allowed to develop until 10.0 hours after the end of egg collection. *lola *mutant and control embryos were collected concurrently. Seven independent sample pairs were used for microarray experiments and three to five for qRT-PCR.

Collected embryos were dechorionated with 50% bleach, rinsed once with 0.1 M Na phosphate pH 7.2, 0.3% Triton X-100, and rinsed twice with sterile water. Total RNA was then extracted with Trizol (Invitrogen, Carlsbad, CA, USA) per the manufacturer's instructions. The final RNA pellet was rinsed twice with 75% ethanol, air-dried for 10 minutes, resuspended in 20 μl RNase-free water at 60°C and stored at -70°C. Each RNA sample for microarrays comprised 20 to 30 μg of total RNA, derived from approximately 300 embryos; samples for RT-PCR were derived from 50 embryos.

### Array methods

Preparation of spotted cDNA arrays bearing the *Drosophila *Gene Collection (release 1 and release 2), RNA-labeling, hybridization, scanning and extraction of feature intensities using GenePix were performed by the Fred Hutchinson Cancer Research Center (FHCRC) Genome Analysis Facility as described elsewhere [32]. Of the seven RNA pairs that were analyzed for each array experiment, in four cases the mutant sample was labeled with Cy3 and control with Cy5; in the remaining three cases the labels were reversed. Microarray data have been deposited to the NCBI GEO database.

### Statistical methods for analysis of array data

Array data analysis was performed with *limma *[34,35] in the R statistical programming language (R Development Core Team) [56]. Briefly, spot intensity data (from GenePix) was read-in using the *limma *function *read.maimages*. No background correction was applied, within-array intensity values were normalized using *print tip loess*, and between-array intensity values were normalized using *scale*. Gene-wise linear models to the normalized intensity ratios were fitted using *limFit *with default parameters. Several statistics, including the moderated t-statistic and the log-odds of differential expression, were calculated for each array probe using *eBayes*. The moderated t-statistics were classified as significant using an adjusted *P*-value of 0.05. Adjustment for multiple testing was done using the Benjamini-Hochberg method for controlling the false discovery rate (limma function *decideTests*, with adjust.method = 'BH'). Spots were excluded from analysis if no corresponding sequence was available from public databases, if FHCRC production notes indicated spot contamination or if GenePix data extraction software failed to locate the probe in any experiment. In total, 10, 376 features were included in the analysis.

### Quantitative real time PCR

Real time PCR was performed on an Applied Biosystems 7300 Real-Time PCR System using Taqman Gene Expression Assays (Applied Biosystems, Foster City, CA, USA). Primer sets were purchased commercially (Applied Biosystems) as follows: *Ribosomal protein L32 *(RPL32; Dm02151827_g1); *Bekka *(Dm02363268_s1); *blistery *(Dm02138346_m1); *CG6070 *(Dm02145281_m1); *charybde *(Dm01802349_m1); *Kruppel target at 95D *(Dm02150605_m1); *Neural Lazarillo *(Dm01844577_g1); *spire *(Dm01811138_m1); *Target of rapamycin *(Dm01843300_g1); and *walrus *(Dm01792969_g1). Gene expression levels were normalized to the endogenous control *RpL32 *using the standard curve method according to the manufacturer's instructions. Normalized gene expression levels of *lola *null mutant samples were measured relative to wild-type control. Measurements were repeated with three to five biological replicates, and each biological replicate was performed with three technical replicates.

### Histochemistry and microscopy

Embryos for *in situ *hybridization and protein immunostaining were prepared and examined by standard methods [26]. Anti-Fasciclin 2 was from the University of Iowa Developmental Studies Hybridoma Bank, the biotin anti-mouse secondary was from Jackson ImmunoResearch (West Grove, PA, USA) and detection was with Vectastain (Vector Labs, Burlingame, CA, USA **{ **Reagents for *in situ *hybridization and alkaline phosphatase detection were from Roche Applied Sciences (Indianapolis, IN, USA).

ISNb phenotype was quantified by labeling stage 17 embryos with anti-Fasciclin 2, using DAB detection, and counting the number of neuromuscular junctions to ventral longitudinal muscles (muscles 7, 6, 13 and 12) in segments A2 to A7 in fillet preparations. Embryo genotypes were determined unambiguously using gratuitous markers. Statistical significance was assessed by t-test; N = approximately 200 hemisegments per dataset.

## Abbreviations

CNS: central nervous system; FHCRC: Fred Hutchinson Cancer Research Center; GFP: green fluorescent protein; PNS: peripheral nervous system; qRT-PCR: quantitative RT-PCR.

## Competing interests

The authors declare that they have no competing interests.

## Authors' contributions

MAG performed the microarray analysis and validation, RK performed the genetic validation and characterization of *spire *expression, EG was responsible for overall design and contributed to interpretation of the results. All three authors contributed to writing of the manuscript and all read and approved its final form.

## Supplementary Material

Additional file 1 **Table S1. Complete list of array features reporting altered RNA level in *lola^ORE76 ^*mutant embryos**. The table lists all probes from the *Drosophila *Gene Collection that reported significantly altered RNA accumulation in *lola *null mutant embryos versus the matched wild-type control (ordered by *P*-value). BLAST analysis was used to assign each probe to the corresponding *Drosophila *gene where possible; these are given by FBgn number and locus name. Fold change is reported as log2 and statistical support is reported as adjusted *P*-value [35]. Note that *lola^ORE76 ^*is a nonsense mutation, and indeed, features corresponding to *lola *isoforms reported only modest changes in transcript level. Unfortunately, expression of *slit*, *robo *and *robo3 *could not be assayed in this experiment as they were either absent from this release of the *Drosophila *Gene Collection or found to be defective during chip preparation. *robo2 *did not show a significant change in RNA accumulation. Click here for file

## References

[B1] Butler SJ Tear G Getting axons onto the right path: the role of transcription factors in axon guidance Development 2007 134 439 448 17185317 10.1242/dev.02762

[B2] Polleux F Ince-Dunn G Ghosh A Transcriptional regulation of vertebrate axon guidance and synapse formation Nat Rev Neurosci 2007 8 331 340 17453014 10.1038/nrn2118

[B3] Simon M Receptor tyrosine kinases: Specific outcomes from general signals Cell 2000 103 13 15 10.1016/S0092-8674(00)00100-8 11051543

[B4] Tanabe Y Jessell TM Diversity and pattern in the developing spinal cord Science 1996 274 1115 1123 10.1126/science.274.5290.1115 8895454

[B5] Ghysen A Jan LY Jan YN Segmental determination in *Drosophila *central nervous system Cell 1985 40 943 948 10.1016/0092-8674(85)90354-X 3886161

[B6] Gould AP White RA Connectin, a target of homeotic gene control in *Drosophila* Development 1992 116 1163 1174 1363542 10.1242/dev.116.4.1163

[B7] Thor S Andersson SG Tomlinson A Thomas JB A LIM-homeodomain combinatorial code for motor-neuron pathway selection Nature 1999 397 76 80 10.1038/16275 9892357

[B8] Thor S Thomas JB The *Drosophila islet *gene governs axon pathfinding and neurotransmitter identity Neuron 1997 18 397 409 10.1016/S0896-6273(00)81241-6 9115734

[B9] Tsuchida T Ensini M Morton SB Baldassare M Edlund T Jessell TM Pfaff SL Topographic organization of embryonic motor neurons defined by expression of LIM homeobox genes Cell 1994 79 957 970 10.1016/0092-8674(94)90027-2 7528105

[B10] Labrador JP O'Keefe D Yoshikawa S McKinnon RD Thomas JB Bashaw GJ The homeobox transcription factor even-skipped regulates netrin-receptor expression to control dorsal motor-axon projections in *Drosophila* Curr Biol 2005 15 1413 1419 10.1016/j.cub.2005.06.058 16085495

[B11] Yang L Garbe DS Bashaw GJ A frazzled/DCC-dependent transcriptional switch regulates midline axon guidance Science 2009 324 944 947 10.1126/science.1171320 19325078 PMC4078765

[B12] Winberg ML Mitchell KJ Goodman CS Genetic analysis of the mechanisms controlling target selection: complementary and combinatorial functions of netrins, semaphorins, and IgCAMs Cell 1998 93 581 591 10.1016/S0092-8674(00)81187-3 9604933

[B13] Kaufmann N Wills ZP Van Vactor D *Drosophila *Rac1 controls motor axon guidance Development 1998 125 453 461 9425140 10.1242/dev.125.3.453

[B14] Wills Z Bateman J Korey CA Comer A Van Vactor D The tyrosine kinase Abl and its substrate enabled collaborate with the receptor phosphatase Dlar to control motor axon guidance Neuron 1999 22 301 312 10.1016/S0896-6273(00)81091-0 10069336

[B15] Wills Z Marr L Zinn K Goodman CS Van Vactor D Profilin and the Abl tyrosine kinase are required for motor axon outgrowth in the *Drosophila *embryo Neuron 1999 22 291 299 10.1016/S0896-6273(00)81090-9 10069335

[B16] Garbe DS Bashaw GJ Axon guidance at the midline: from mutants to mechanisms Crit Rev Biochem Mol Biol 2004 39 319 341 10.1080/10409230490906797 15763708

[B17] Crowner D Madden K Goeke S Giniger E Lola regulates midline crossing of CNS axons in *Drosophila* Development 2002 129 1317 1325 11880341 10.1242/dev.129.6.1317

[B18] Madden K Crowner D Giniger E *lola *has the properties of a master regulator of axon-target interactions for SNb motor axons of *Drosophila* Dev Biol 1999 213 301 313 10.1006/dbio.1999.9399 10479449

[B19] Brenman JE Gao FB Jan LY Jan YN Sequoia, a tramtrack-related zinc finger protein, functions as a pan-neural regulator for dendrite and axon morphogenesis in *Drosophila* Dev Cell 2001 1 667 677 10.1016/S1534-5807(01)00072-7 11709187

[B20] Spletter ML Liu J Su H Giniger E Komiyama T Quake S Luo L Lola regulates *Drosophila *olfactory projection neuron identity and targeting specificity Neural Dev 2007 2 14 10.1186/1749-8104-2-14 17634136 PMC1947980

[B21] Edwards AC Zwarts L Yamamoto A Callaerts P Mackay TF Mutations in many genes affect aggressive behavior in *Drosophila *melanogaster BMC Biol 2009 7 29 10.1186/1741-7007-7-29 19519879 PMC2707370

[B22] Mackay TF Heinsohn SL Lyman RF Moehring AJ Morgan TJ Rollmann SM Genetics and genomics of *Drosophila *mating behavior Proc Natl Acad Sci USA 2005 102 Suppl 1 6622 6629 15851659 10.1073/pnas.0501986102 PMC1131870

[B23] Morozova TV Anholt RR Mackay TF Transcriptional response to alcohol exposure in *Drosophila melanogaster* Genome Biol 2006 7 R95 10.1186/gb-2006-7-10-r95 17054780 PMC1794562

[B24] Yamamoto A Zwarts L Callaerts P Norga K Mackay TF Anholt RR Neurogenetic networks for startle-induced locomotion in *Drosophila melanogaster* Proc Natl Acad Sci USA 2008 105 12393 12398 10.1073/pnas.0804889105 18713854 PMC2527922

[B25] Bass BP Cullen K McCall K The axon guidance gene *lola *is required for programmed cell death in the *Drosophila *ovary Dev Biol 2007 304 771 785 10.1016/j.ydbio.2007.01.029 17336958 PMC1905497

[B26] Goeke S Greene EA Grant PK Gates MA Crowner D Aigaki T Giniger E Alternative splicing of *lola *generates 19 transcription factors controling axon guidance in *Drosophila* Nat Neurosci 2003 6 917 924 10.1038/nn1105 12897787

[B27] Ohsako T Horiuchi T Matsuo T Komaya S Aigaki T *Drosophila lola *encodes a family of BTB-transcription regulators with highly variable C-terminal domains containing zinc finger motifs Gene 2003 311 59 69 12853139 10.1016/s0378-1119(03)00554-7

[B28] Zhang W Wang Y Long J Girton J Johansen J Johansen KM A developmentally regulated splice variant from the complex lola locus encoding multiple different zinc finger domain proteins interacts with the chromosomal kinase JIL-1 J Biol Chem 2003 278 11696 11704 10.1074/jbc.M213269200 12538650

[B29] Cavarec L Jensen S Casella JF Cristescu SA Heidmann T Molecular cloning and characterization of a transcription factor for the *copia *retrotransposon with homology to the BTB-containing lola neurogenic factor Mol Cell Biol 1997 17 482 494 8972229 10.1128/mcb.17.1.482 PMC231773

[B30] Manseau LJ Schupbach T *cappuccino *and *spire*: two unique maternal-effect loci required for both the anteroposterior and dorsoventral patterns of the *Drosophila *embryo Genes Dev 1989 3 1437 1452 10.1101/gad.3.9.1437 2514120

[B31] Gates MA From transcription to axon guidance: Uncovering downstream effectors of *longitudinals lacking *in *Drosophila melanogaster* PhD thesis 2008 University of Washington and Fred Hutchinson Cancer Research Center, Program in Molecular and Cell Biology

[B32] Pierce SB Yost C Anderson SA Flynn EM Delrow J Eisenman RN *Drosophila *growth and development in the absence of *dMyc *and *dMnt* Dev Biol 2008 315 303 316 10.1016/j.ydbio.2007.12.026 18241851 PMC2322934

[B33] Stapleton M Liao G Brokstein P Hong L Carninci P Shiraki T Hayashizaki Y Champe M Pacleb J Wan K Yu C Carlson J George R Celniker S Rubin GM The *Drosophila *gene collection: identification of putative full-length cDNAs for 70% of *D*. *melanogaster *genes Genome Res 2002 12 1294 1300 10.1101/gr.269102 12176937 PMC186637

[B34] Smyth GK Speed T Normalization of cDNA microarray data Methods 2003 31 265 273 10.1016/S1046-2023(03)00155-5 14597310

[B35] Smyth GK Linear models and empirical Bayes methods for assessing differential expression in microarray experiments Stat Appl Genet Mol Biol 2004 3 Article 3 10.2202/1544-6115.1027 16646809

[B36] Benjamini Y Hochberg Y Controlling false discovery rate: A practical and powerful approach to multiple testing J Royal Statistical Soc B 1995 57 289 300

[B37] Huang DW Sherman BT Lempicki RA Systematic and integrative analysis of large gene lists using DAVID bioinformatics resources Nat Protoc 2009 4 44 57 19131956 10.1038/nprot.2008.211

[B38] Quinlan ME Heuser JE Kerkhoff E Mullins RD *Drosophila *Spire is an actin nucleation factor Nature 2005 433 382 388 10.1038/nature03241 15674283

[B39] Rosales-Nieves AE Johndrow JE Keller LC Magie CR Pinto-Santini DM Parkhurst SM Coordination of microtubule and microfilament dynamics by *Drosophila Rho1*, *Spire *and *Cappuccino* Nat Cell Biol 2006 8 367 376 10.1038/ncb1385 16518391 PMC1997291

[B40] Theurkauf WE Premature microtubule-dependent cytoplasmic streaming in *cappuccino *and *spire *mutant oocytes Science 1994 265 2093 2096 10.1126/science.8091233 8091233

[B41] Schumacher N Borawski JM Leberfinger CB Gessler M Kerkhoff E Overlapping expression pattern of the actin organizers *Spir-1 *and *formin-2 *in the developing mouse nervous system and the adult brain Gene Expr Patterns 2004 4 249 255 10.1016/j.modgep.2003.11.006 15053972

[B42] Giniger E Tietje K Jan LY Jan YN *lola *encodes a putative transcription factor required for axon growth and guidance in Drosophila Development 1994 120 1385 1398 8050351 10.1242/dev.120.6.1385

[B43] Seeger M Tear G Ferres-Marco D Goodman CS Mutations affecting growth cone guidance in *Drosophila*: Genes necessary for guidance towards or away from the midline Neuron 1993 10 409 426 10.1016/0896-6273(93)90330-T 8461134

[B44] Kolodziej PA Timpe LC Mitchell KJ Fried SR Goodman CS Jan LY Jan YN *frazzled *encodes a *Drosophila *member of the DCC immunoglobulin subfamily and is required for CNS and motor axon guidance Cell 1996 87 197 204 10.1016/S0092-8674(00)81338-0 8861904

[B45] Hu S Sonnenfeld M Stahl S Crews ST Midline Fasciclin: a *Drosophila *Fasciclin-I-related membrane protein localized to the CNS midline cells and trachea J Neurobiol 1998 35 77 93 10.1002/(SICI)1097-4695(199804)35:1<77::AID-NEU7>3.0.CO;2-8 9552168

[B46] Chiba A Snow P Keshishian H Hotta Y Fasciclin III as a synaptic target recognition molecule in *Drosophila* Nature 1995 374 166 168 10.1038/374166a0 7877688

[B47] Shishido E Takeichi M Nose A *Drosophila *synapse formation: regulation by transmembrane protein with Leu-rich repeats, CAPRICIOUS Science 1998 280 2118 2121 10.1126/science.280.5372.2118 9641918

[B48] Sanchez D Ganfornina MD Torres-Schumann S Speese SD Lora JM Bastiani MJ Characterization of two novel lipocalins expressed in the *Drosophila *embryonic nervous system Int J Dev Biol 2000 44 349 359 10949044

[B49] Ferres-Marco D Gutierrez-Garcia I Vallejo DM Bolivar J Gutierrez-Avino FJ Dominguez M Epigenetic silencers and Notch collaborate to promote malignant tumours by Rb silencing Nature 2006 439 430 436 10.1038/nature04376 16437107

[B50] Zheng L Carthew RW Lola regulates cell fate by antagonizing *Notch *induction in the *Drosophila *eye Mech Dev 2008 125 18 29 10.1016/j.mod.2007.10.007 18053694 PMC2782576

[B51] Vizcarra CL Kreutz B Rodal AA Toms AV Lu J Zheng W Quinlan ME Eck MJ Structure and function of the interacting domains of Spire and Fmn-family formins Proc Natl Acad Sci USA 2011 108 11884 11889 10.1073/pnas.1105703108 21730168 PMC3141961

[B52] Hoffmann FM *Drosophila abl *and genetic redundancy in signal transduction TIGS 1991 7 351 355 10.1016/0168-9525(91)90254-f 1820686

[B53] Kuzina I Song JK Giniger E How Notch establishes longitudinal axon connections between successive segments of the Drosophila CNS Development 2011 138 1839 1849 10.1242/dev.062471 21447553 PMC3074455

[B54] Luo L Liao J Jan LY Jan YN Distinct morphogenetic functions of similar small GTPases *Drosophila *Drac1 is involved in axonal outgrowth and myoblast fusion Genes Dev 1994 8 1787 1802 10.1101/gad.8.15.1787 7958857

[B55] Giniger E How do Rho family GTPases direct axon growth and guidance? A proposal relating signaling pathways to growth cone mechanics Differentiation 2002 70 385 396 10.1046/j.1432-0436.2002.700801.x 12366376

[B56] The R Project for Statistical Computing http://www.R-project.org

